# Structural Characterization of the SARS-CoV-2 Replication and Transcription Complex

**DOI:** 10.1063/4.0001136

**Published:** 2025-10-27

**Authors:** Jennifer L Warnock, Sharique Khan, Wellington Leite, Susan Tsutakawa, Gregory Hura, Hugh O'Neill

**Affiliations:** 1 Oak Ridge National Laboratory, Oak Ridge, TN, USA; 2 Lawrence Berkeley National Laboratory, Berkeley, CA, USA

## Abstract

At around 30 kb, the SARS-CoV-2 genome is one of the largest viral genomes among RNA viruses. As such, the replication machinery must be prepared to quickly and efficiently replicate this genome in order for the viral infection to effectively persist. In this study, we used several techniques to study the SARS-CoV-2 replication and transcription complex (RTC). We assessed complex stoichiometry and binding kinetics via mass photometry (MP) and analytical ultracentrifugation (AUC). We also employed small-angle x-ray scattering (SAXS), size-exclusion chromatography-coupled small-angle x-ray scattering (SEC-SAXS), and small-angle neutron scattering (SANS) in order to characterize the structure and protein-protein interactions of the SARS-CoV-2 replication complex proteins with each other and with RNA. This data has allowed us to gain further understanding of the formation and structure of the RTC.

